# A new modular framework for high-level application development at HEPS

**DOI:** 10.1107/S160057752301086X

**Published:** 2024-02-01

**Authors:** Xiaohan Lu, Yaliang Zhao, Hongfei Ji, Yi Jiao, Jingyi Li, Nan Li, Cai Meng, Yuemei Peng, Daheng Ji, Yuanyuan Wei, Haisheng Xu, Weimin Pan, Gang Xu

**Affiliations:** a Institute of High Energy Physics, Chinese Academy of Sciences, Beijing, People’s Republic of China; Australian Synchrotron, Australia

**Keywords:** high-level application, *Pyapas*, physical quantities

## Abstract

As a representative of the fourth-generation light sources, the High Energy Photon Source (HEPS) in Beijing, China, utilizes a multi-bend achromat lattice to obtain an approximately 100 times emittance reduction compared with third-generation light sources. New technologies bring new challenges to operate the storage ring. In order to meet the beam commissioning requirements of HEPS, a new framework for the development of high-level applications (HLAs) has been created. The key part of the new framework is a dual-layer physical module to facilitate the seamless fusion of physical simulation models with the real machine, allowing for fast switching between different simulation models to accommodate the various simulation scenarios. As a framework designed for development of physical applications, all variables are based on physical quantities. This allows physicists to analytically assess measurement parameters and optimize machine parameters in a more intuitive manner. To enhance both extensibility and adaptability, a modular design strategy is utilized, partitioning the entire framework into discrete modules in alignment with the requirements of HLA development. This strategy not only facilitates the independent development of each module but also minimizes inter-module coupling, thereby simplifying the maintenance and expansion of the entire framework. To simplify the development complexity, the design of the new framework is implemented using Python and is called *Python-based Accelerator Physics Application Set* (*Pyapas*). Taking advantage of Python’s flexibility and robust library support, we are able to develop and iterate quickly, while also allowing for seamless integration with other scientific computing applications. HLAs for both the HEPS linac and booster have been successfully developed. During the beam commissioning process at the linac, *Pyapas*’s ease of use and reliability have significantly reduced the time required for the beam commissioning operators. As a development framework for HLA designed for the new-generation light sources, *Pyapas* has the versatility to be employed with HEPS, as well as with other comparable light sources, due to its adaptability.

## Abstract 2

The development of *Pyapas*, a new framework for high-level applications at the High Energy Photon Source, Beijing, China, is discussed. *Pyapas* enables the easy integration of simulation models with real machine operations, simplifies development complexity, and has been shown to effectively reduce time for beam commissioning.

## 1. Introduction

The High Energy Photon Source (HEPS) (Jiao *et al.*, 2018 ▸; Jiao & Pan, 2022 ▸) is a fourth-generation light source with an ultralow emittance of about 35 pm rad, currently under construction in Beijing, China. The HEPS accelerator complex comprises a linac and a booster as injector, a 1.3 km, 6 GeV storage ring and three transfer lines (Jiao *et al.*, 2020 ▸; Meng *et al.*, 2020 ▸; Peng *et al.*, 2020 ▸; Guo *et al.*, 2020 ▸). To achieve an ultralow emittance, HEPS adopts a compact multi-bend achromat (MBA) lattice, coupled with the extensive use of combined-function magnets. This approach results in several challenges to the design, commissioning and operation of the light source, *e.g.* pronounced cross-talk effects between magnets, stringent dynamic and static error tolerances, necessitating an efficient and stable framework for the development of high-level applications (HLAs).

The HLAs focus on implementing complex algorithms to analyse, measure and control physical parameters. These include tuning the magnetic field of magnets instead of their current, getting beam orbit from beam-position-monitor readings for correction, tune optimization and calculating optical parameters based on the response matrix, *etc*. The development of HLAs is necessary for every accelerator facility. With the increasing complexity of accelerator facilities, the approach of developing applications individually based on specific requirements becomes more and more difficult in terms of meeting the development demands. Furthermore, during the beam commissioning, conducting extensive simulations based on physical models for predicting beam behaviour is crucial. Conventional approaches usually require offline simulations that use real-time magnet parameters with subsequent adjustments based on these simulation results, which would notably reduce the efficiency of the beam commissioning process. As a result, implementing online simulations and adjusting parameters based on simulation results in real time has become a widely accepted practice in HLA development across different laboratories (Zhukov *et al.*, 2019 ▸; Corbett *et al.*, 2003 ▸; Portmann *et al.*, 2005 ▸; Stevani *et al.*, 2016 ▸; Yang *et al.*, 2013 ▸). Consequently, there has been an emergence of many systematic development frameworks for HLAs. For example, the XAL (Zhukov *et al.*, 2019 ▸) developed by SNS based on Java offers a series of functions and tools for HLA development, and the MML (MATLAB Middle Layer) (Corbett *et al.*, 2003 ▸; Portmann *et al.*, 2005 ▸) developed by SLAC integrates many commonly used tools and greatly facilitates the setup and control of a storage ring. XAL incorporates an online model for model-based control, and MML employs *Accelerator Toolbox* (*AT*) (Terebilo, 2001 ▸) for the same purpose. These integrated development frameworks provide extremely convenient development tools for the construction of HLAs, ensuring good consistency and stability in applications development.

However, these integrated frameworks, with their highly interconnected and interdependent functions, may not be very flexible and transportable in some scenarios. Particularly when new requirements emerge or when functional updates and iterations are necessary, considerable time and effort are required. For HEPS, due to the complicated beam dynamics, various simulation tools are used for simulation studies (Xu *et al.*, 2023 ▸). During the design and research phase, *MADX*, *elegant*, *AT*, *BMAD*, *etc*. were utilized to investigate different problems (Jiao *et al.*, 2021 ▸; Wang *et al.*, 2023 ▸; Duan *et al.*, 2021 ▸; He *et al.*, 2021 ▸). Similar situations will likely be encountered during the beam commissioning and operation. Moreover, with an MBA lattice, the number of magnets in fourth-generation light sources has increased by an order of magnitude compared with existing third-generation light sources. This means that the variables to be controlled have increased by one or two orders of magnitude. The error tolerances of the fourth-generation light sources are also tighter due to the ultralow emittance and stronger magnetic fields. Therefore, higher control precision and faster response times need to be considered in the HLA development. In the past few years there has been an increase in new solutions to address the mentioned requirements, such as more efficient communication tools, more convenient interface development tools, and more reliable database systems. Integrating new technologies can effectively fulfil our new requirements for HLA development.

Based on the above considerations, we have developed a brand new framework named *Pyapas* (*Python-based Accelerator Physics Application Set*) for HEPS, by incorporating design experiences from various HLA development frameworks and referring to the design logic of XAL and PyDM (https://slaclab.github.io/pydm/) with new technologies. *Pyapas* is designed with a modular concept, by dividing it into several modules based on the functionalities required for HLA development: graphic interface module, dual-layer physical module, communication module, client-server module, database module, and pre-development module. Each module is designed individually according to its functionality, ensuring its completeness and independence. We have established protocols for inter-module communication and provided concise interfaces to minimize coupling between the modules. This modular design greatly enhances the extensibility and maintainability of the development framework. Upgrading or even replacing a single module will not significantly impact other modules or the developed applications. As an HLA development framework oriented towards physics, the core of *Pyapas* is the dual-layer physical module which can set up the connection from various simulation models to the real machine. Also, a more efficient communication system has been designed to communicate with the real machine.

The modular structure design and the dual-layer physical module have significantly improved the extensibility and usability of the HLA development framework. To further reduce the complexity of HLA development we implemented the *Pyapas* design in Python, which has concise and readable syntax, robust libraries and strong community support. We basically completed the HLA development for the linac, booster, transfer line and the storage ring (Lu *et al.*, 2021 ▸). The development of HLAs based on *Pyapas* has used remarkably concise code to accomplish relatively complex measurement applications (Lu *et al.*, 2023*a* ▸), and *Pyapas* has been used in commissioning the linac and booster successfully.

The outline of the paper is as follows. In Section 2 an overview of the design of *Pyapas* and its core design principles are discussed. Section 3 introduces how to implement the design with Python to make the framework more reliable and extensible. In Section 4 we briefly introduce the development of the HLAs based on *Pyapas* and their application to the beam commissioning of the linac and booster. Conclusions are given in Section 5.

## 2. Framework design

The development of HLAs is a huge project, especially for a facility like HEPS with a large number of elements. A significant amount of optimization applications need to be prepared for optimizing beam parameters. The improvement on application development efficiency and application quality must be considered. Improving development efficiency is crucial, especially under conditions of limited human resources. The design of a HLA framework itself is one of the most effective strategies to improve development efficiency – with a well designed framework the HLA developers can save a lot of time. A good framework should have comprehensive functions, easy-to-use interfaces and excellent stability. To achieve these desirable attributes, a modular design approach is adopted.

**Figure 1:**
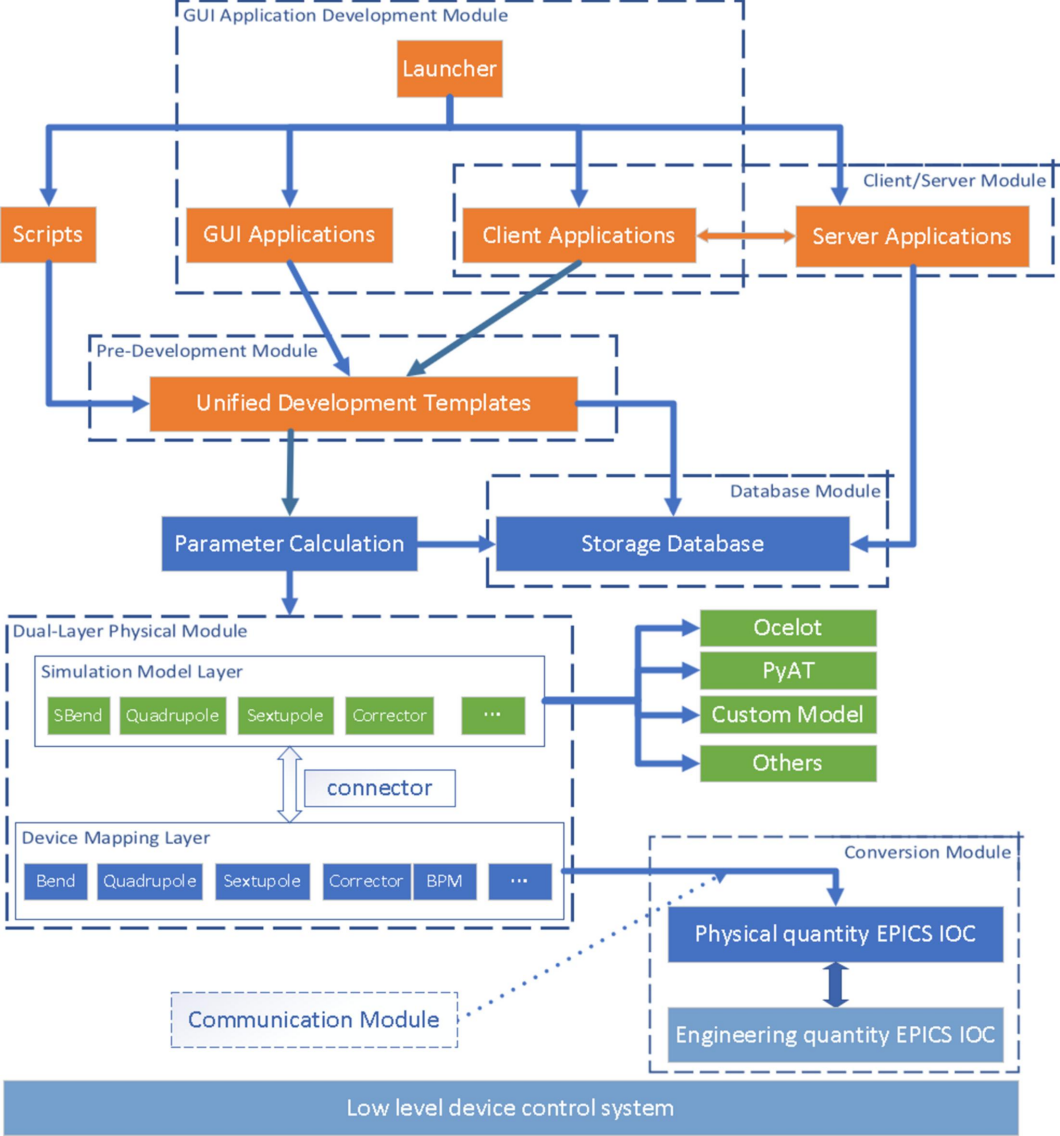
The new modular framework of HLAs.

As shown in Fig. 1, the framework supports different types of HLAs, scripts, GUI applications and server-based applications. There are some unified development templates for rapid application development, which can reduce the time spent on application development and improve the stability of the applications. These developed applications can invoke simulation models through the dual-layer physical module and read real-time machine settings for online calculations. Notably, the calculated results such as beam trajectory and Twiss parameters can also be compared with the real-time measurement data. Furthermore, the flexibility of these applications is evident as they can seamlessly switch between different simulation models online through the dual-layer physical module to perform different tasks and cross check. Additionally, with the database module the integration with the database is streamlined, and all the applications can easily access and store the required data.

In accordance with the specific requirements of the HLAs, we have divided the functions involved into several distinct modules: the graphical user interface (GUI) application development module, the physical module, the communication module, the server module, the database module and the pre-development module. As shown in Fig. 1 ▸, with the dual-layer physical module as the core, each module is designed and developed independently. A straightforward interface is provided according to the needs of physical beam commissioning. The physics developers do not need to understand the technical details behind, rather only simple invocation is needed. This strategy not only facilitates the integration of comprehensive functions but also helps in creating user-friendly interfaces, ensuring exceptional stability. Through modular design, each component of the framework can be developed, tested and refined independently, fostering a robust and efficient development environment that meets the highest standards of quality and performance.

The distinctive aspect of HLAs is its involvement with various physical algorithms and the online calculation of machine parameters. Therefore, when designing a new HLA framework, it is crucial to consider not only the provision of basic development functions but also the integration of dependable physical algorithms and robust online simulation models. As such, the design of an online physical calculation module should be the primary focus.

**Figure 2:**
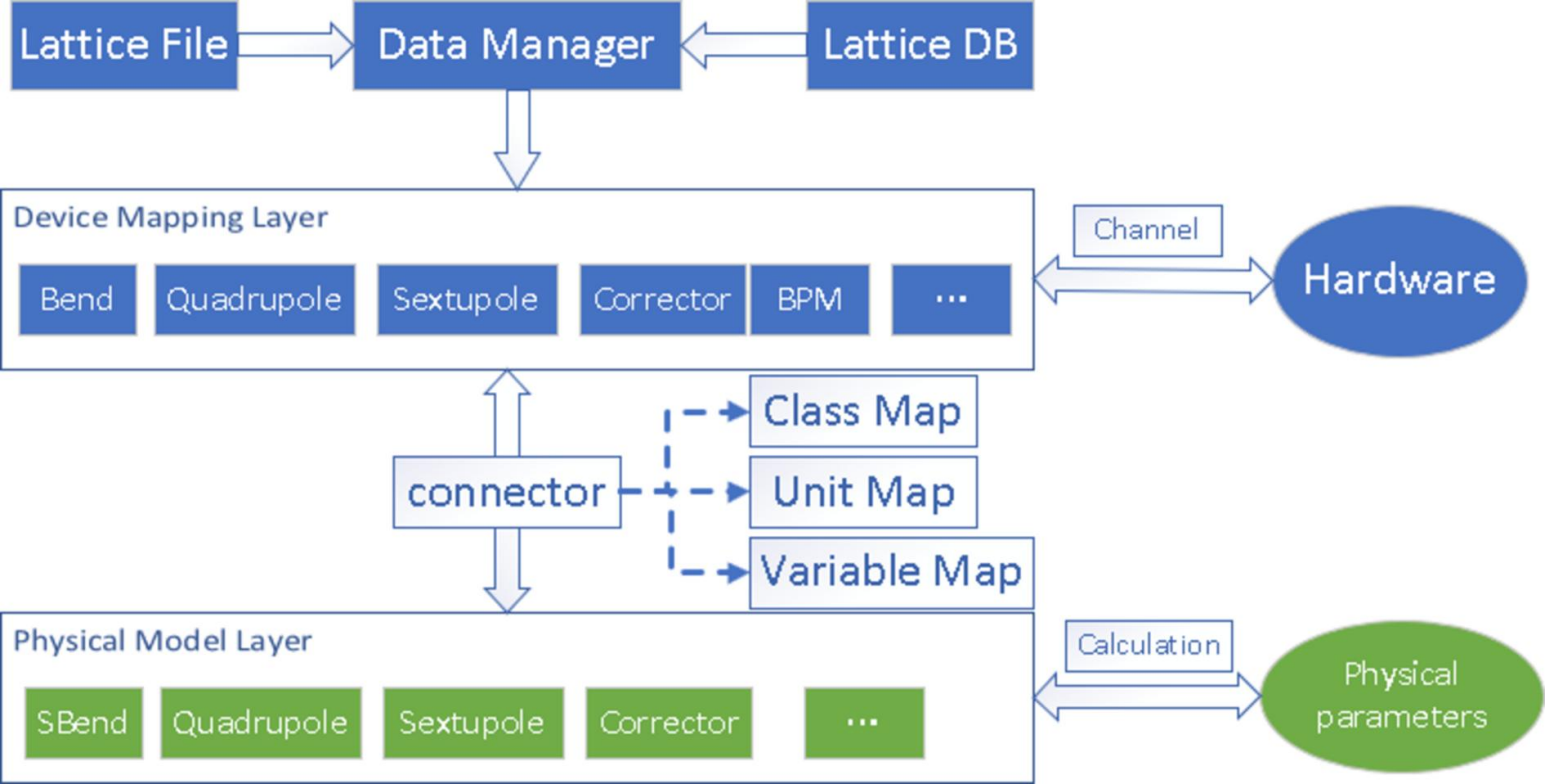
The dual-layer physical module.

As mentioned earlier, integrated development frameworks typically include a specialized simulation model designed for a specific device. If alternative models are required, significant adaptation work is necessary. Fourth-generation light sources, represented by HEPS, involve numerous physical processes with higher simulation accuracy requirements. Different models are often used for simulation studies during the design phase. In the beam commissioning phase, we also hope to use different models for online simulation and control of different physical processes of the beam. To meet this requirement, a dual-layer physical module has been designed, as shown in Fig. 2 ▸. The first layer is the device mapping layer, defining various types of accelerator elements, including combined elements. The element class contains various basic parameters such as length, position, physical quantities and related channels for communication with the actual machine. The second layer is a simulation model layer, which includes mathematical models of a series of components. The first and second layers are independent of each other, with their mutual communication facilitated by a connection class. The connection class does not require any complicated code, other than just a few simple dictionaries that define the correspondence between the two layers, the class names, the names of the parameters and the conversion of units. The interface of the element mapping layer will correctly pass parameters to the simulation model layer for simulation calculations according to the corresponding relationship. Based on this design, changing the corresponding parameters in the connection class allows us to quickly switch between different simulation models online. This decoupled dual-layer design can greatly increase its scalability. Defining or calling different simulation models only requires writing the corresponding relationship in the connector. It is worth mentioning here that, to ensure the stability and reliability of online programs, the invocation of simulation models is done directly by calling the corresponding calculation functions or element calculation classes.

The dual-layer physical module can easily implement online physical parameter calculations. However, there is still a problem that must be considered during the implementation process. Physical parameter calculations usually involve physical quantities such as magnetic fields, energy, angles, *etc*., while the control quantities of actual accelerator elements are engineering quantities, such as current. This means that the data read from the actual accelerator elements cannot be directly used for physical parameter calculations. If each HLA can directly read physical quantities from a low-level control system, the difficulty of the development will be further reduced and the data of HLA will be more consistent and intuitive.

**Figure 3:**
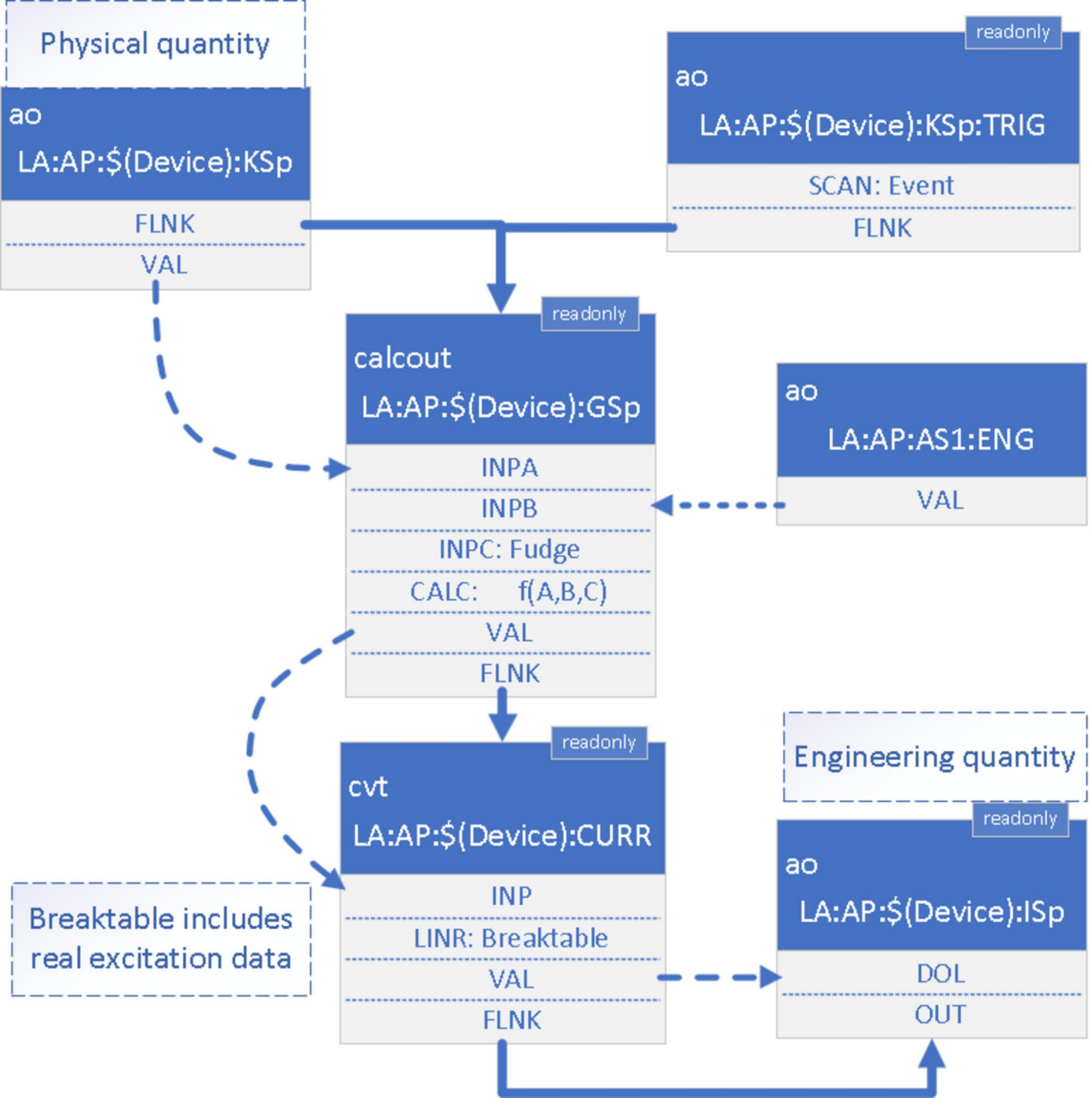
The conversion process from physical quantities to engineering quantities.

To solve this problem, another design principle based on physical quantities is proposed. All the variables of HLA should be physical quantities and a conversion system using *Experimental Physics and Industrial Control System* (*EPICS*) is established to help HLA control the real machine with physical quantities. As an example, Fig. 3 ▸ shows the process converting from physical quantity *K* (focusing strength of a quadrupole) to engineering quantity *I* (power supply current of the quadrupole), based on measured excitation data to perform mutual conversions between physical and engineering quantities. The excitation curve measured for a quadrupole magnet establishes the relationship between the magnetic field gradient and current. So the first step of the conversion system is to convert the value *K* to the magnetic field gradient *G*, as shown in equation (1), 

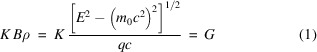

where *B*ρ is the magnetic rigidity, *E* is the total energy of the particle, *q* is the charge of the particle, *m*_0_ is the rest mass and *c* is the speed of light; afterwards, the value *G* is converted to the current *I* using interpolation based on the excitation curve. To perform the conversion process, a new *EPICS* record type ‘cvt’ has been developed. The conversion system is deployed in the control system, and it works in conjunction with the HLA based on physical quantities, perfectly realizing direct control of the actual machine based on physical quantities, greatly facilitating the commissioning process for the operators.

## 3. Implementation with Python

The systematic framework design can assist developers in quickly developing and deploying their applications, aiming to minimize the time cost of HLA development. As all the design discussed in the last section strives to enhance the adaptability and extensibility of the HLA development, the choice of a programming language emerges as a vital consideration. Python, standing as one of the most popular scripting languages globally, naturally comes to the forefront. Its popularity is attributed to its ease of learning, stability and robust library support, which has fostered its growing prominence in the particle accelerator area. Recognizing these advantages, we chose to implement the new framework in pure Python, leveraging its capabilities to further enhance the efficiency and effectiveness of the HLA development process. As mentioned, this brand new framework was named *Pyapas*.

In the last section, the key design principles have been established: modular design, dual-layer physical module, physical quantity-based strategy. A conversion system has been developed and deployed in the control system to help *Pyapas* accomplish physical quantity-based control. For the dual-layer physical module, the road map is very clear – a couple of Python classes were defined to map to the element of the real accelerator. For the physical calculation layer, *OCELOT* (Agapov *et al.*, 2014 ▸), which is developed based on pure Python, is used as the primary simulation model. Other simulation models, such as *PyAT* (Rogers *et al.*, 2017 ▸) and specifically developed models, are now also available. We developed ‘kick–drift–kick’ models for simulating combined magnets and other special elements.

Additionally, to improve the completeness and usability of *Pyapas*, we utilized Python’s extensive library ecosystem to incorporate efficient communication module, advanced interface development tools, reliable server and database modules, as well as pre-developed module.

**Figure 4:**
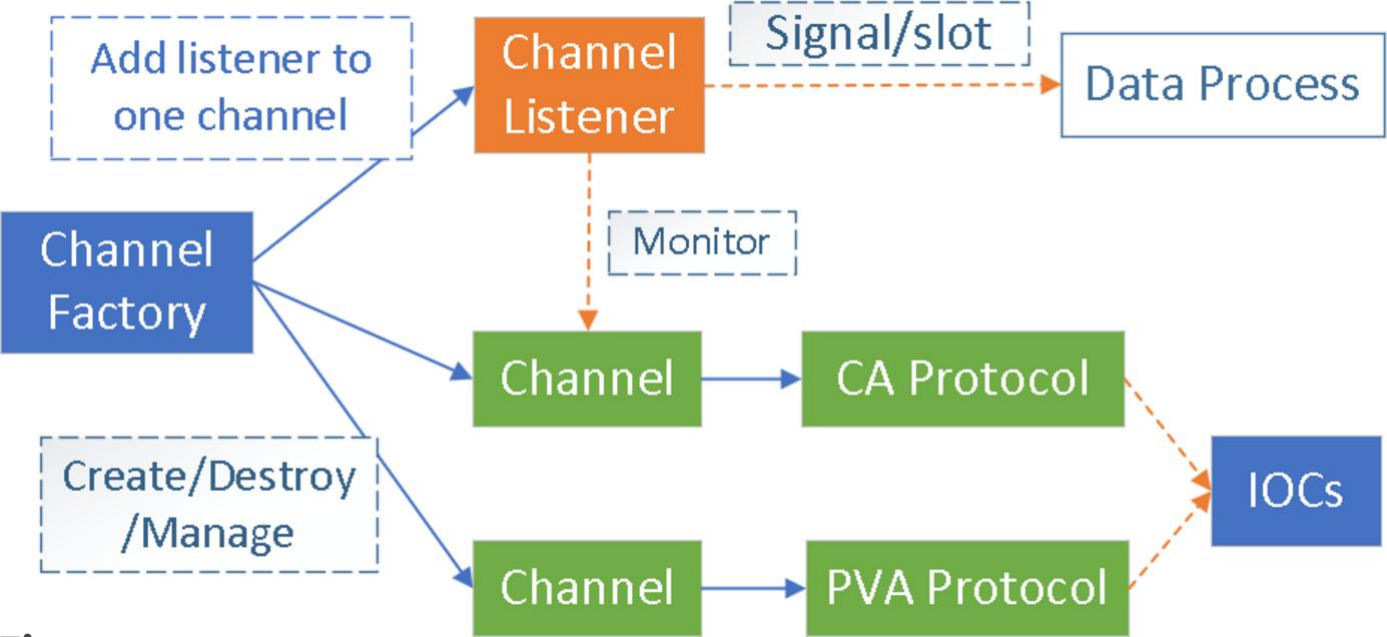
The communication module design.

As discussed earlier, a high-efficiency communication system is required to address the challenges of managing a large number of control variables while maintaining effective communication. As the low-level control system of HEPS is based on EPICS, the communication module of *Pyapas* needs to communicate with the EPICS system. To ensure the stability and consistency of the HLAs during communication operations, we have designed a comprehensive communication module for the development of HLAs based on the factory pattern, singleton pattern and *PyQt*’s (https://wiki.python.org/moin/PyQt) signal-slot mechanism, as shown in Fig. 4 ▸. The creation and destruction of all channels are managed by the ‘ChannelFactory’ class. The factory pattern can decouple the creation and usage process, reduce code duplication, and maximize the avoidance of errors caused by creation logic errors, thereby greatly enhancing stability. In addition to the traditional ‘factory pattern’, we have developed a more specialized factory pattern called the ‘singleton factory pattern’. In the same commissioning application, if the factory class encounters a second instantiation request, it will automatically return the reference to the existing instance, rather than creating a new one. This avoids creating multiple factory classes in the same application and causing repeated channel connections, which may exhaust network resources in a serious scenario. At the same time, to more conveniently establish monitoring of a specific *EPICS* channel and obtain the real-time value of the channel, a listener class in conjunction with *Qt*’s signal-slot mechanism was designed. This class can automatically establish non-blocking connections from the channel to the receiving function, monitoring changes in the channel value.

In the development of HLAs, the most time-consuming aspect is the development of the GUI. In the interface development module, *Pyapas* uses *PyQt* as the interface construction tool and introduces *QtDesigner* as the main interface design tool. *QtDesigner* provides basic interface widgets for drag-and-drop style interface design. The generated GUI files can be directly loaded into specific applications, greatly reducing the time cost of interface development. However, basic widgets are not sufficient to meet the needs of complex application development. To further reduce development time, we made customizations for common interactive needs in physical commissioning and loaded them as plugins into *QtDesigner*. Developers can perform drag-and-drop development to complete the design of relatively complex interactive interfaces. The interface development module contains numerous plugins to meet different needs, such as embedding the *pyqtgraph* and *matplotlib* drawing tools into *QtDesigner* and widgets with hardware communication capabilities.

In the development of the server module, we encapsulated a simple and easy-to-use server class based on *XML Remote Procedure Call* (*xmlRPC*) and *multicast DNS* (*mDNS*) technology. *xmlRPC* serves as a protocol that uses XML to encode its calls with HTTP as a transport mechanism. This technology facilitates the communication between different systems, allowing for a seamless exchange of information and commands. *mDNS* operates as a protocol within the IP network to resolve hostnames to IP addresses within small networks without requiring a dedicated DNS server. This technology is particularly beneficial in local network environments, where it can facilitate easy discovery of services and hosts. When these two technologies are combined within a server module, they create a powerful and flexible module that can greatly simplify the development process. Developers only need to inherit the ‘Server’ class and invoke the registration function to create a fully functional server application. This approach not only reduces the development time but also ensures that the application adheres to best practices in terms of scalability and performance.

In the database module, we adopted Object Relation Mapping technology and further encapsulated it. Developers only need to call the corresponding function to pass in the data list, the corresponding table name or filter conditions, and they can implement basic functions such as data insertion, deletion and search. This meets general application development needs, greatly reduces time cost and minimizes the likelihood of errors.

In order to unify the development style, save the development time and enhance application stability, we created a pre-development module that offers standardized templates that simplify application development. It provides pre-defined programming structures and coding standards, promoting a uniform and efficient approach. This consistency not only speeds up development but also improves stability. The template includes best programming practices and robust error handling to minimize common coding pitfalls and runtime errors, thereby enhancing application stability. These templates have undergone a thorough review and testing process to ensure the reliability and stability of the code blocks, which in turn reinforces the overall integrity of the application.

We implemented the framework design of *Pyapas* in Pure Python. Each module was independently designed and encapsulated with a simple and easy-to-use interface. The design of each module is premised on stability and ease of use. Moreover, based on these modules, we also designed various useful tools to help physics developers quickly develop HLAs, such as a multi-threaded parameter scanner, multichannel data fetcher, lattice manager, *etc*., ultimately forming a comprehensive HLA development platform. Based on *Pyapas*, we have completed the HLA development of the linac and booster and successfully applied it to actual beam commissioning.

## 4. Application in HEPS beam commissioning

**Figure 5:**
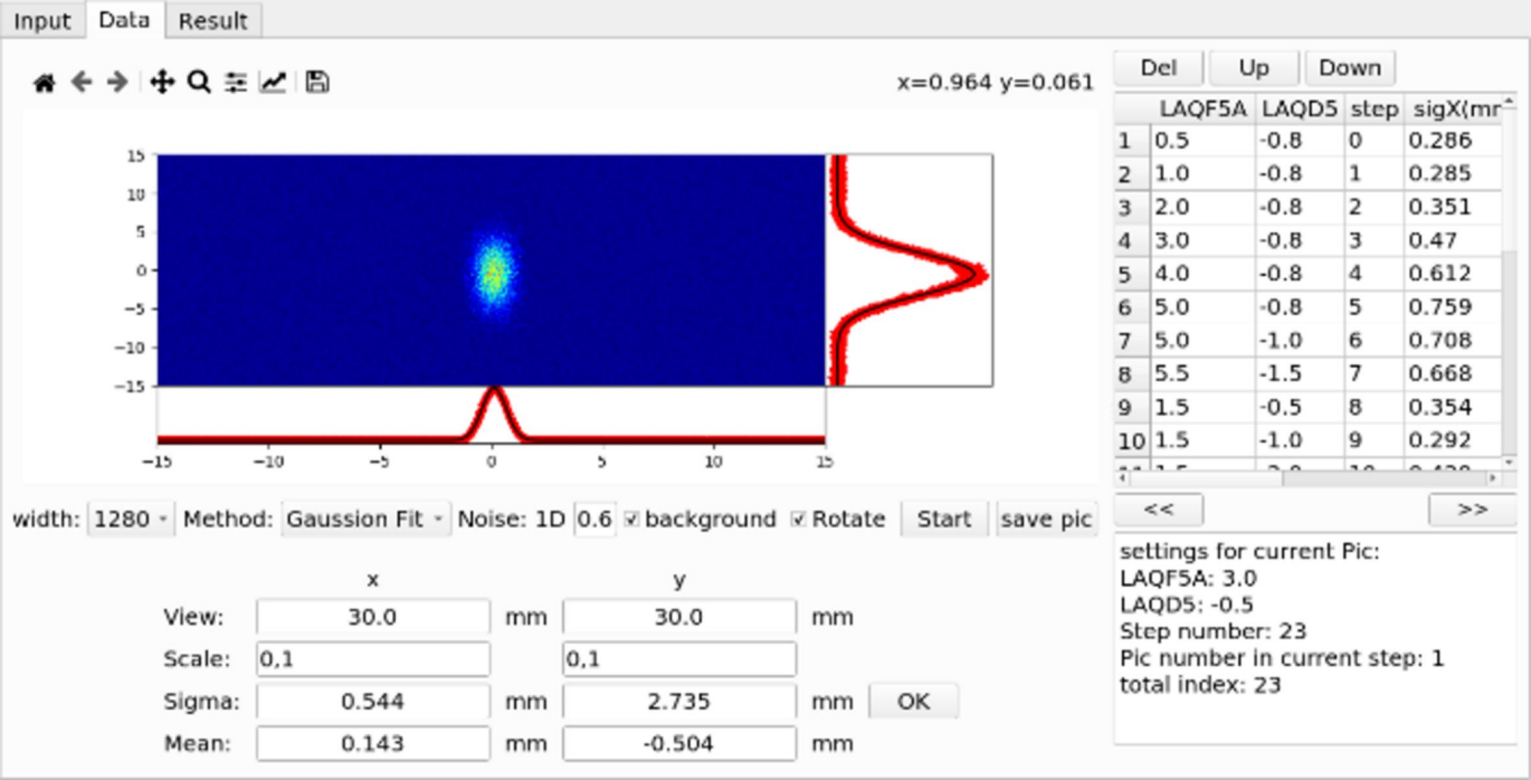
The emittance measurement application.

**Figure 6:**
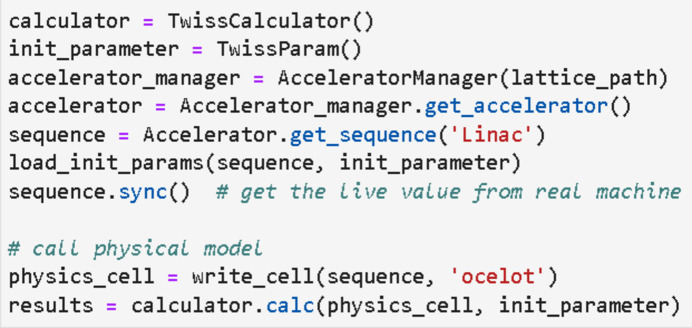
Example of an online Twiss calculation.

We have applied the HLAs based on *Pyapas* to the beam commissioning of the HEPS injector. On 9 March 2023 we began the beam commissioning of the linac (Meng *et al.*, 2023*a* ▸,*b* ▸). To meet the beam commissioning needs of the linac, we have developed applications such as beam-based alignment, emittance measurement application, phase scan application, orbit correction application, *etc.* (Zhao *et al.*, 2023 ▸). These applications need to interact with the actual machine and perform online physical parameter calculations, requiring a good interactive interface, data storage functions, *etc*. Developed based on *Pyapas*, these applications implemented all necessary functions with just a few lines of code. Taking the emittance measurement application as an example, the quadrupole scanning method (Zhao *et al.*, 2018 ▸) is used to obtain the emittance and Twiss parameter. To develop this application, the core tasks are GUI development, data acquisition and Twiss parameter calculation. As shown in Fig. 5 ▸, it is very easy to design a GUI with the GUI development module. With the communication module, less than five lines of code are needed to set up a monitor to get the beam profile data from the diagnostic system. Then it only takes about ten lines of code to compute the real-time machine Twiss parameters, as shown in Fig. 6 ▸. The developer spends most time focused on result calculation and error analysis.

The pre-developed modules of *Pyapas* greatly reduce the difficulty of developing online measurement applications. Meanwhile, we used a relatively accurate simulation model, which makes the results more credible. For the results of emittance measurement, at the high bunch charge mode with a single bunch charge of 7 nC, the measured horizontal emittance is about 56.4 nm rad, satisfying the design target of below 70 nm rad.

**Figure 7:**
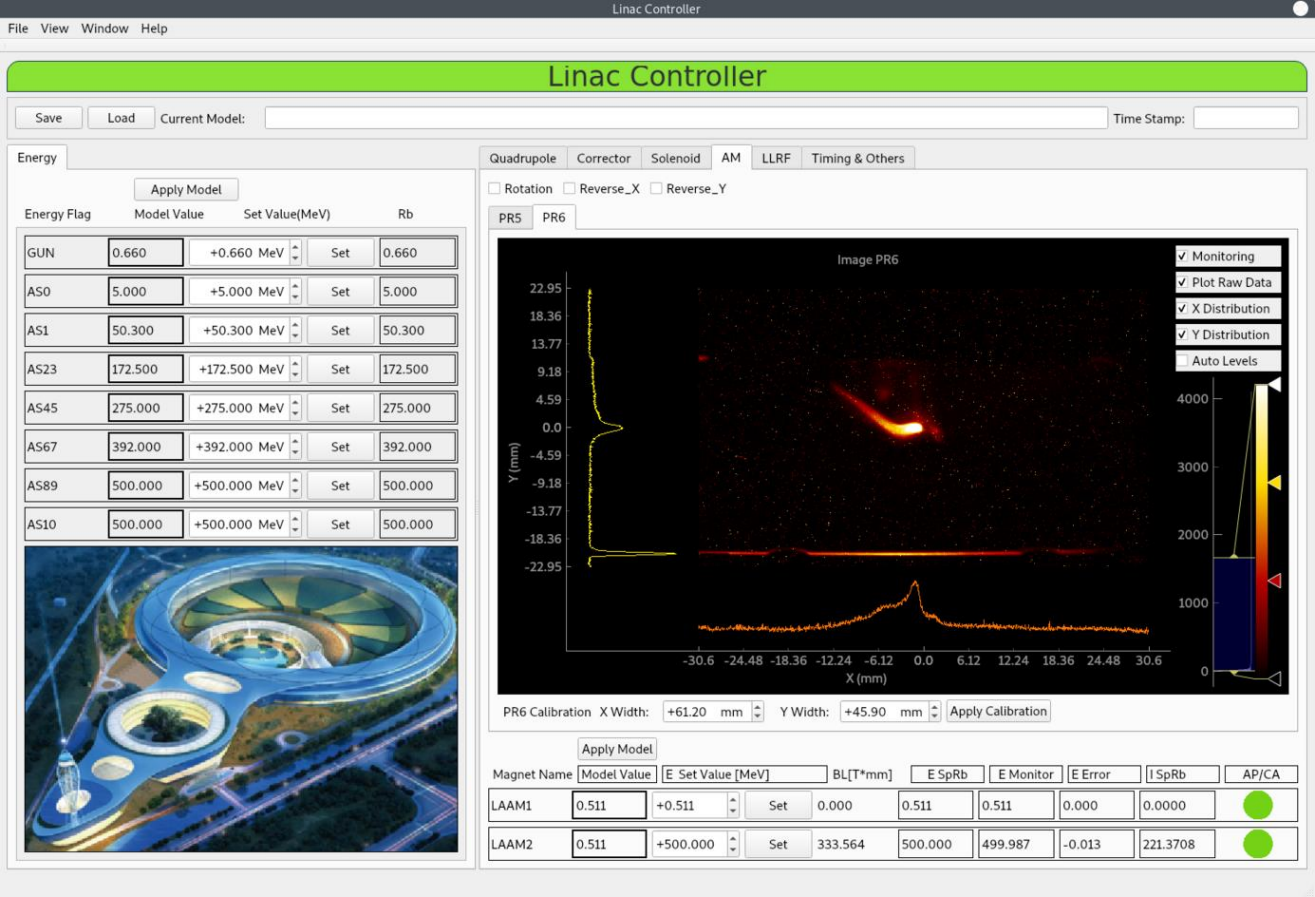
Linac Controller based on physical quantities.

In addition to measurement applications that include algorithms, *Pyapas* can enable even more straightforward and efficient development for control monitoring applications. Taking the Linac Controller application as an example, as shown in Fig. 7 ▸, the custom widgets with channel properties enable us to complete all functions through drag-and-drop development in *QtDesigner*. Loading the generated user interface file requires less than ten lines of code, greatly simplifying the development process.

Beam commissioning of the booster began in late July of 2023 and is to be started in 2024 for the storage ring. Before the commissioning, we have developed corresponding HLAs based on *Pyapas*, including global orbit correction, local orbit correction, dispersion measurement, physics-based control programs, chromaticity measurement, first-turn beam analysis, *etc*. All applications have been developed and tested in multiple rounds on a virtual accelerator (Lu *et al.*, 2023*b* ▸), which was also developed based on *Pyapas*.

## 5. Conclusion

To meet the beam commissioning requirements of the fourth-generation synchrotron light source HEPS, we have designed a brand-new HLA development framework and implemented it in Python, named *Pyapas*. The use of *Pyapas* can significantly reduce the development time cost. After two years of development, *Pyapas* now has almost all the necessary functions, and all HLAs of HEPS are developed based on *Pyapas*. With limited human resources, we have quickly completed the development of about 30 applications and successfully applied them to the beam commissioning of the HEPS injector. *Pyapas* is a feature-complete, clear-structured and easy-to-use framework for HLA development. Its well designed structure ensures high reliability and maintainability. The modular design makes it easy to extend the framework, and the dual-layer physical module allows for quick switching between different simulation models for online calculations. For example, the communication module can be expanded to support other control systems, such as *Tango* (https://tango-controls.readthedocs.io/). This can be achieved by adding the appropriate code to the communication module to make interface calls. All applications developed with *Pyapas* have a universal design that allows easy adaptation to a new accelerator. This process simply involves preparing a special configuration file for the new accelerator, containing critical details such as component layout, connection channel names and database settings. Once this configuration file is in place, applications can be easily applied to the new accelerator. *Pyapas* features remarkable extensibility, robust stability and superior portability, making it a versatile solution not only for different light sources but also for a range of other large-scale experimental facilities.

In the near future, it is planned to develop machine-learning-related applications based on *Pyapas*. The use of machine-learning algorithms is increasingly common in particle accelerator research, with numerous successful applications. Effective data acquisition and utilization is a critical step in both training and deploying these algorithms. Inadequate data quality or insufficient marked data can result in model training failures. *Pyapas* took into account machine learning as a future application from the outset. The design based on physical quantities can accurately correlate physical measurements with data and store them in real time within a database, which will greatly simplify the model training process for machine-learning studies. Along with other software systems and solutions (Liu *et al.*, 2022 ▸; Li *et al.*, 2023 ▸; Zhang *et al.*, 2023 ▸; Dong *et al.*, 2022 ▸) designed for the big data acquisition and analysis tasks at HEPS beamlines, a whole Python-based automatic and intelligent scientific software ecosystem is emerging to facilitate cutting-edge scientific discovery at HEPS.
